# Supplementary material for: Enhancing Hepatocellular Carcinoma Surveillance: Comparative Evaluation of AFP, AFP-L3, DCP and Composite Models in a Biobank-Based Case-Control Study
Source: Cancers (Basel). 2025 Jul 18;17(14):2390. doi: 10.3390/cancers17142390 (PMC12293724; doi:10.3390/cancers17142390)
Supplement: Supplementary file 1 [file cancers-17-02390-s001.zip › Supplementary Table S1.pdf]

Supplementary Table S1. Characteristics of patients with hepatocellular carcinoma

	HCC (n=165)
**Tumor size**, *median (IQR), mm*	43.0 (9.0–162.0)
**Number of tumors**, *n (%)*	
• *1*	94 (57.3)
• *2*	20 (12.2)
• *3*	16 (9.8)
• *Multiple*	30 (18.3)
• *Diffuse*	4 (2.4)
**Lymph node involvement**, *n (%)*	26 (15.9)
**Portal vein thrombosis**, *n (%)*	30 (18.3)
**Vascular invasion**, *n (%)*	19 (11.6)
**Extrahepatic metastasis**, *n (%)*	7 (4.3)
**BCLC stage**, *n (%)*	
• *0*	10 (6.1)
• *A*	54 (32.9)
• *B*	67 (40.9)
• *C*	24 (14.6)
• *D*	9 (5.5)

BCLC, Barcelona Clinic Liver Cancer; HCC: Hepatocellular carcinoma; IQR, interquartile range.
