# Supplementary material for: Enhancing Hepatocellular Carcinoma Surveillance: Comparative Evaluation of AFP, AFP-L3, DCP and Composite Models in a Biobank-Based Case-Control Study
Source: Cancers (Basel). 2025 Jul 18;17(14):2390. doi: 10.3390/cancers17142390 (PMC12293724; doi:10.3390/cancers17142390)
Supplement: Supplementary file 1 [file cancers-17-02390-s001.zip › Supplementary Table S2.pdf]

Supplementary Table S2. Pairwise comparisons of serologic biomarkers and composite models

Parameter	p-value
AFP	Healthy controls vs CLD: <0.001*
	Healthy controls vs HCC: <0.001*
	CLD vs HCC: <0.001*
AFP-L3	Healthy controls vs CLD: <0.001*
	Healthy controls vs HCC: <0.001*
	CLD vs HCC: <0.001*
DCP	Healthy controls vs CLD: 0.337
	Healthy controls vs HCC: <0.001*
	CLD vs HCC: <0.001*
GALAD	Healthy controls vs CLD: <0.001*
	Healthy controls vs HCC: <0.001*
	CLD vs HCC: <0.001*
GAAP	Healthy controls vs CLD: <0.001*
	Healthy controls vs HCC: <0.001*
	CLD vs HCC: <0.001*
ASAP	Healthy controls vs CLD: 0.007*
	Healthy controls vs HCC: <0.001*
	CLD vs HCC: <0.001*
aMAP	Healthy controls vs CLD: <0.001*
	Healthy controls vs HCC: <0.001*
	CLD vs HCC: <0.001*
Doylestown	Healthy controls vs CLD: <0.001*
	Healthy controls vs HCC: <0.001*
	CLD vs HCC: <0.001*

AFP: Alpha-fetoprotein; AFP-L3: Lens culnaris agglutinin-reactive alpha-fetoprotein; CLD: Chronic liver disease, DCP: Des-gamma-carboxy prothrombin, HCC: Hepatocellular carcinoma
* indicates p value < 0.05
