# Supplementary material for: Enhancing Hepatocellular Carcinoma Surveillance: Comparative Evaluation of AFP, AFP-L3, DCP and Composite Models in a Biobank-Based Case-Control Study
Source: Cancers (Basel). 2025 Jul 18;17(14):2390. doi: 10.3390/cancers17142390 (PMC12293724; doi:10.3390/cancers17142390)
Supplement: Supplementary file 1 [file cancers-17-02390-s001.zip › Supplementary Table S3.pdf]

Supplementary Table S3. Univariate Binary Logistic Regression Analysis of Variables Associated with Hepatocellular Carcinoma

	**Univariate**			
	Beta-coefficient	***OR***	***95% CI***	***p-value***
**Age**	*0.092*	*1.096*	*1.072-1.120*	*<0.001**
**Male gender**	*0.819*	*2.269*	*1.469-3.504*	*<0.001**
**Body-mass index**	*-0.018*	*0.982*	*0.941-1.025*	*0.410*
**Diabetes mellitus**	0.462	1.587	1.050-2.399	0.028*
**Hypertension**	0.688	1.990	1.298-3.053	0.002*
**Hyperlipidemia**	0.607	1.835	0.925-3.640	0.083
**Viral etiology (vs. non-viral etiology)**	-0.279	0.756	0.498-1.148	0.190
**Cirrhosis**	2.194	8.973	5.020-16.038	<0.001*
**CTP score**	0.444	1.558	1.349-1.800	<0.001*
**Esophageal varices**	0.595	1.813	1.189-2.764	0.006*
**Variceal bleeding**	0.978	2.658	1.202-5.880	0.016*
**Creatinine**	0.437	1.548	0.799-2.998	0.196
**Platelet count**	-0.001	0.999	0.997-1.001	0.329
**AFP**	0.048	1.049	1.024-1.075	<0.001*
**AFP-L3**	0.060	1.061	1.045-1.077	<0.001*
**DCP**	0.032	1.033	1.018-1.048	<0.001*
**GALAD**	0.717	2.048	1.772-2.367	<0.001*
**ASAP**	0.845	2.327	1.964-2.758	<0.001*
**GAAP**	0.788	2.200	1.880-2.574	<0.001*
**aMAP**	0.085	1.089	1.063-1.115	<0.001*
**Doylestown**	5.553	258.026	98.786-673.959	<0.001*

AFP: Alpha-fetoprotein; AFP-L3: Lens culnaris agglutinin-reactive alpha-fetoprotein; CI: Confidence Interval; CTP: Child-Turcotte-Pugh, DCP: Des-gamma-carboxy prothrombin, OR: Odds ratio
* indicates p value <0.05
